# Classification and Extraction of Resting State Networks Using Healthy and Epilepsy fMRI Data

**DOI:** 10.3389/fnins.2016.00440

**Published:** 2016-09-27

**Authors:** Svyatoslav Vergun, Wolfgang Gaggl, Veena A. Nair, Joshua I. Suhonen, Rasmus M. Birn, Azam S. Ahmed, M. Elizabeth Meyerand, James Reuss, Edgar A. DeYoe, Vivek Prabhakaran

**Affiliations:** ^1^Medical Physics, University of Wisconsin-MadisonMadison, WI, USA; ^2^Radiology, University of Wisconsin-MadisonMadison, WI, USA; ^3^Prism Clinical Imaging, Inc.,Elm Grove, WI, USA; ^4^Psychiatry, University of Wisconsin-MadisonMadison, WI, USA; ^5^Neurological Surgery, University of Wisconsin-MadisonMadison, WI, USA; ^6^Biomedical Engineering, University of Wisconsin-MadisonMadison, WI, USA; ^7^Radiology, Medical College of WisconsinMilwaukee, WI, USA; ^8^Cell Biology, Neurobiology and Anatomy, Medical College of WisconsinMilwaukee, WI, USA; ^9^Biophysics, Medical College of WisconsinMilwaukee, WI, USA; ^10^Psychology, University of Wisconsin-MadisonMadison, WI, USA

**Keywords:** resting state fMRI, resting state networks, independent component analysis, machine learning, classification

## Abstract

Functional magnetic resonance imaging studies have significantly expanded the field's understanding of functional brain activity of healthy and patient populations. Resting state (rs-) fMRI, which does not require subjects to perform a task, eliminating confounds of task difficulty, allows examination of neural activity and offers valuable functional mapping information. The purpose of this work was to develop an automatic resting state network (RSN) labeling method which offers value in clinical workflow during rs-fMRI mapping by organizing and quickly labeling spatial maps into functional networks. Here independent component analysis (ICA) and machine learning were applied to rs-fMRI data with the goal of developing a method for the clinically oriented task of extracting and classifying spatial maps into auditory, visual, default-mode, sensorimotor, and executive control RSNs from 23 epilepsy patients (and for general comparison, separately for 30 healthy subjects). ICA revealed distinct and consistent functional network components across patients and healthy subjects. Network classification was successful, achieving 88% accuracy for epilepsy patients with a naïve Bayes algorithm (and 90% accuracy for healthy subjects with a perceptron). The method's utility to researchers and clinicians is the provided RSN spatial maps and their functional labeling which offer complementary functional information to clinicians' expert interpretation.

## Introduction

With the use of task and resting state fMRI (rs-fMRI), much progress has been made describing typical and atypical human brain activity at the group level. The main clinical applications of task fMRI have been in mapping motor, language, and memory networks in presurgical planning of patients with brain tumors, vascular lesions, and epilepsy (Detre, 2004; Laufs and Duncan, 2007; Zijlmans et al., 2007; Greicius, 2008). An active area of research is the use of rs-fMRI for presurgical mapping of functional tissue in individual patients, analogous to task fMRI (Lang et al., 2014).

Rs-fMRI measures synchronous activations between regions that are spatially distinct, occurring while a subject is not constrained to perform a task and is given no stimulus. It has demonstrated reliable, large-scale coherent networks (Damoiseaux et al., 2006; Shehzad et al., 2009; Van Dijk et al., 2010; Song et al., 2012), and importantly for neurosurgery, a sensorimotor network and various language networks have been consistently replicated (Lang et al., 2014). Even during this period of rest it has been shown that functional networks, also utilized by the brain while performing a task, are continuously and dynamically active (Biswal et al., 1995; Fox and Raichle, 2007; Smith et al., 2009). Rs-fMRI has been shown to be able to identify several key networks in patients (Kokkonen et al., 2009; Liu et al., 2009; Shimony et al., 2009). Leading researchers in the field have shown that rs-fMRI can provide spatial maps that closely correspond to task activation maps (Kokkonen et al., 2009; Smith et al., 2009) and intraoperative cortical stimulation maps (Liu et al., 2009; Zhang et al., 2009; Mitchell et al., 2013). It can provide valuable presurgical information in many patients who cannot perform traditional task-based fMRI, and has several advantages over task fMRI. Rs-fMRI scans offer quick scan time capturing all of the networks at once (≈7 min as compared to the time required for many scans during task fMRI presurgical mapping), have no task performance requirements (often many trials are required to adequately perform a task), and can be acquired alongside routine clinical MR scanning of the patient (Zhang et al., 2009; Lee et al., 2013; Lang et al., 2014).

Several methods have been developed to study such neural connectivity: voxel based (Ashburner and Friston, 2000), region of interest (ROI) based (Poldrack, 2007), graph theory (Van Den Heuvel and Hulshoff Pol, 2010), independent component analysis (ICA; Calhoun et al., 2001; van de Ven et al., 2004; Beckmann et al., 2005), and machine learning methods (Dosenbach et al., 2010; Cohen et al., 2011). The work described in this article combines two established methods, ICA and machine learning, to develop an automatic process of extracting and identifying (classifying) network maps in the context of clinical workflow. For the task of localizing functional network spatial maps, the method of ICA is a natural choice (De Martino et al., 2007; Tohka et al., 2008; Smith et al., 2009). ICA spatial maps have been consistently replicated in many studies (Smith et al., 2009; Biswal et al., 2010; Allen et al., 2011) and shown to closely correspond to task activation maps (Kokkonen et al., 2009; Liu et al., 2009; Smith et al., 2009; Zhang et al., 2009). A recent study by Mitchell et al. (2013) used machine learning to extract spatial network maps from rs-fMRI scans and showed a close correspondence between these maps and electrocortical stimulation maps for the language and motor networks in epilepsy patients.

A study by Salimi-Khorshidi et al. (2014), relevant to this article's classification task, presented excellent performance (over 95% accuracy) in the task of distinguishing between noisy and true signal rs-fMRI IC maps. This showed that resting state ICA classification is not only feasible but can be highly accurate. For specific component labeling, Demertzi et al. (2014) used ICA and a univariate template matching method with an additional step of “neuronality” classification to label IC maps into specific network components from a 10-component model but without evaluating its performance.

**Figure 1:**
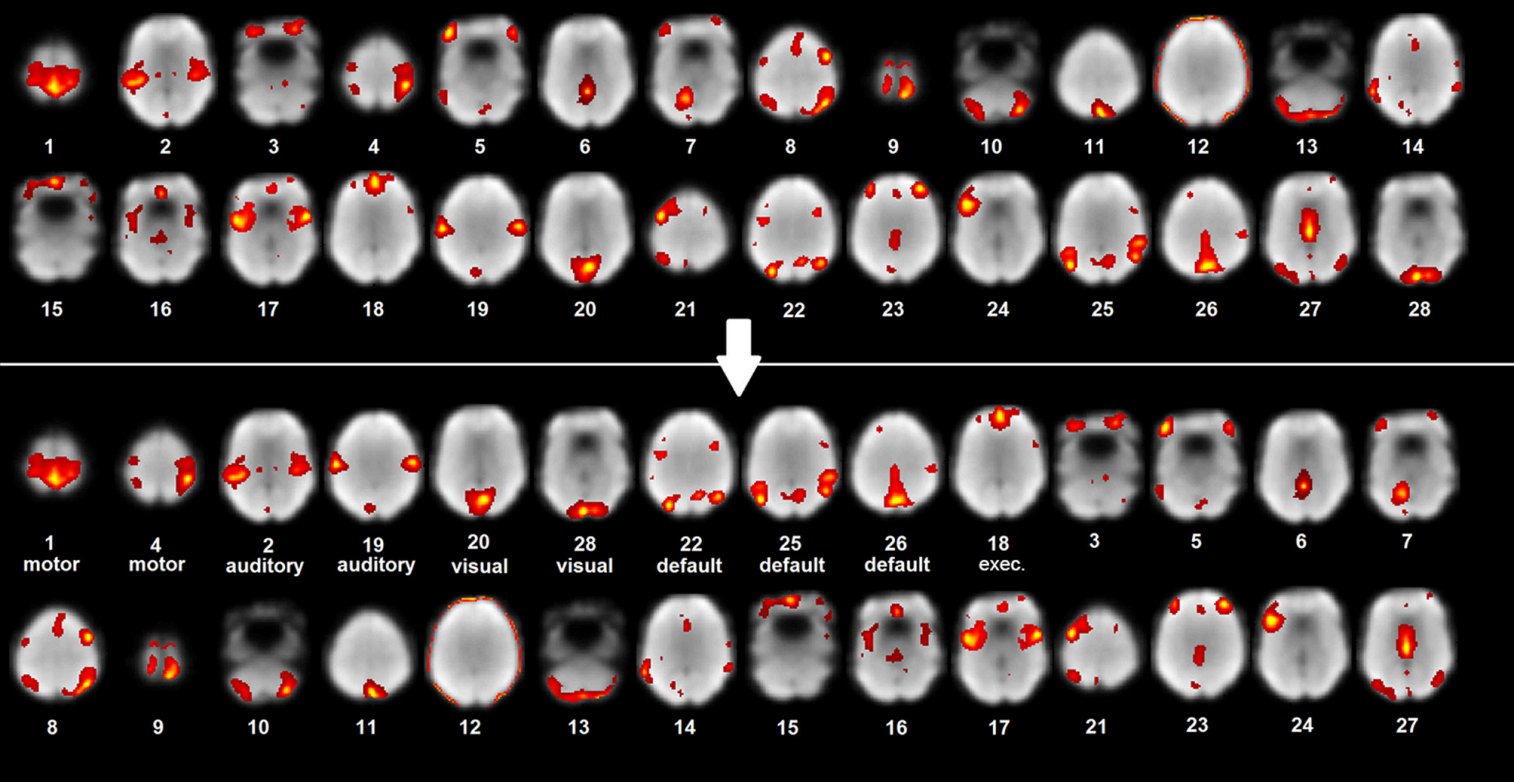
**ICA map labeling and organization**. Example patient unlabeled and labeled ICA maps with rest scan underlay (most representative axial slices shown).

ICA is a data-driven method which uses no a priori information about the brain and has been a popular approach in the analysis of fMRI data (Salimi-Khorshidi et al., 2014). It has the advantage of not requiring a priori, outside knowledge like functional ROI atlases as in seed based analysis, or parameter and measure selections as in graph theory analysis and it can be used complementarily with machine learning. Another difficulty of seed-based correlation mapping, not present in ICA, is that sometimes it is necessary to manually adjust the coordinates of a seed to see a specific network (Zhang et al., 2009). The spatial maps output from ICA have a clear functional and anatomical interpretation: they are the anatomical locations of brain tissue which act synchronously and with the same activity pattern. One difficulty in the process of localizing network maps using ICA is that it outputs many unordered spatial maps (see Figure 1) which requires time consuming interpretation and labeling by a scientist or clinician that manages them (Zhang et al., 2009).

The motivation for developing this classification method was to create a clinical tool that can aid in the presurgical mapping workflow by labeling and organizing the output ICA information. This work's method provides a fast way to extract, coarsely interpret and organize the raw output spatial maps in a standardized, algorithmic way (Figure 1). With respect to the study by Demertzi et al. (2014), this work adds the investigation of the performance of different, complementary multivariate machine learning algorithms for IC spatial map labeling and the evaluation of the labeling method. The potential and accuracy of the method was tested for feasibility using a preliminary set of ICA resting state networks (RSN; auditory, visual, default-mode, sensorimotor, and executive control) from epilepsy patients. Although not functionally complete, these networks are clinically important since their preservation is valuable for minimizing post treatment or resection deficits. To improve clinical utility, language and memory networks must be included (which are partly captured by the fronto-parietal IC maps of Smith et al., 2009). The focus of the paper was the epilepsy population and a healthy subject dataset was used to gain some context for which to compare the epilepsy classifier performance. Machine learning was applied to the task of classifying the extracted spatial maps because of its robustness, ability to handle very high dimensional data and impressive performance (Schölkopf and Smola, 2002; Ben-Hur et al., 2008; Pereira et al., 2009; Meier et al., 2012; Vergun et al., 2013). The extraction and classification method was tested for feasibility on rs-fMRI data from epilepsy patients to investigate its performance on a patient population which benefits from fMRI presurgical mapping.

## Methods

### Participants

Rs-fMRI data from 30 healthy individuals and 23 epilepsy patients, who underwent fMRI as part of presurgical planning, were collected at the University of Wisconsin Hospital and Clinics. The studies were conducted in accordance with protocols (H2008-0142, H2013-1559) approved by the Health Sciences Institutional Review Boards at UW Madison and written, informed consent from all healthy subjects and epilepsy patients was obtained.

### Data sets

The analyses in this work were performed on two datasets separately: healthy subjects and epilepsy patients. The same preprocessing and machine learning algorithms were applied to both datasets independently, and no information from one group was used in the analysis of the other group. Note that the study is focused on the single patient level prediction of ICs and a group level investigation (normal vs. epilepsy discrimination) was not performed.

Data set 1 consisted of 30 healthy subjects (age 18–67, mean = 39.5, 16 M/14 F) and data set 2 consisted of 23 epilepsy patients (age 18–63, mean = 36.5, 10 M/13 F). The patients had no gross structural abnormalities and the resultant brain registration to standard space was satisfactory. All but one of the epilepsy patients were on an epileptic medication routine (one or more drug) during the time that scans were acquired. Most patients were using levetiracetam and zonisamide in combination with other epileptic medications. See Table 1 for a detailed description of the 23 patients (age, gender, epilepsy type, lesion type, epilepsy focus are listed).

**Table 1:** **Epilepsy patient characteristics**.

	**Age, Gender**	**Epilepsy type**	**Specific lesion type**	**(Temporal vs. extra T.)**	**Location**
Patient 1	42, F	Complex partial that can secondarily generalize	Mesial temporal sclerosis	Temporal	Left
Patient 2	34, F	Complex partial, rare secondary generalization	Mesial temporal sclerosis	Temporal	Left
Patient 3	39, M	Partial with secondary generalization	Mesial temporal sclerosis	Temporal	Left
Patient 4	53, M	Partial with complex partial and secondary generalization	Mesial temporal sclerosis	Temporal	Left
Patient 5	53, F	Partial with rare secondary generalization	Parietal cortical dysplasia	Parietal	Right
Patient 6	51, M	Complex partial	Mesial temporal sclerosis	Temporal	Left
Patient 7	25, F	Partial with secondary generalization	Left temporal hypometabolism	Temporal	Left
Patient 8	42, F	Partial	Asymmetry of right temporal lobe	Temporal	Right
Patient 9	28, F	Complex partial with rare secondary generalization	Mesial temporal sclerosis	Temporal	Left
Patient 10	44, M	Complex partial	L temporal cavernoma, 3 mm diam.	Temporal	Left
Patient 11	33, M	Partial with secondary generalization	R > L fronto-temporal polymicrogyria	Fronto-Temporal	Right
Patient 12	19, F	Partial with secondary generalization	Mesial temporal sclerosis	Temporal	Left
Patient 13	26, F	Partial, localization related	Mesial temporal sclerosis	Temporal	Right
Patient 14	31, F	Partial	Partial seizures	Temporal	Left
Patient 15	34, M	Partial with secondary generalization	Frontal lobe encephalomalacia	Frontal	Right
Patient 16	27, F	Simple partial, complex partial	Cystic temporal Encephalomalacia	Temporal	Right
Patient 17	37, F	Partial and partial complex, one that secondarily generalized	Mesial temporal sclerosis	Temporal	Right
Patient 18	53, M	Partial, associated with impairment in consciousness	Mesial temporal atrophy	Temporal	Right
Patient 19	41, M	No EEG correlation	R Temporal neoplasm, 4 mm diam	Temporal	Right
Patient 20	63, F	Partial complex	Gliosis likely post-traumatic	Temporal	Left
Patient 21	25, F	Partial complex	L temporal astrocytoma, 3 mm diam.	Temporal	Left
Patient 22	18, M	Partial localization related with intractable epilepsy	May be cortical irritability, genetic	Occipital	Left
Patient 23	21, M	Generalized epilepsy	Unknown	General	General

### Data acquisition

Resting data were acquired with GE MR750 3T scanners using an axial echo planar imaging (EPI) pulse sequence. The images of healthy subjects consisted of 231 continuous resting state volumes (matrix size = 64 × 64; 40 axial slices; 3.75 × 3.75 × 4 mm; TR 2.6s; TE 0.022s), and for epilepsy patients of 150 volumes (matrix size = 64 × 64; 28 axial slices; 3.75 × 3.75 × 5 mm, TR 2s; TE 0.03s). Subjects and patients were given ear plugs and asked to keep their eyes closed but remain awake and alert during the scan. For spatial normalization and localization, a T1-weighted high resolution anatomical image was acquired using a gradient echo pulse sequence (healthy subjects: IR-prepared FSPGR, matrix size = 256 × 256, 156 axial slices, 1 × 1 × 1 mm, Flip Angle 12°, TR 8.16s, TE 3.18s, TI.45s; epilepsy patients: IR-prepared FSPGR, matrix size = 256 × 256, 140 axial slices, 1 × 1 × 1.2 mm, Flip Angle 12°, TR 8.68s, TE 3.46s, TI 0.45s).

### Data preprocessing

**Figure 2:**
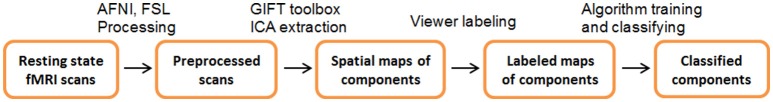
**Extraction and Classification process**. Flowchart of the steps involved in ICA map extraction and classification.

Data were preprocessed using AFNI (http://afni.nimh.nih.gov/afni, version: AFNI_2011_12_21_1014) and FSL (http://www.fmrib.ox.ac.uk/fsl, version: v5.0) open source software. To be consistent with the RSN independent component (IC) templates used in one classification algorithm, the method's preprocessing followed the standard procedure reported by Allen et al. (2011). The steps were: (1) discarding the first four resting scan volumes to remove T1 equilibrium effects, (2) motion and slice-timing correction, (3) skull stripping, (4) spatial normalization to standard Montreal Neurological Institute (MNI) brain space with resampling to 3 × 3 × 3 mm voxels, (5) spatial smoothing with a Gaussian kernel with a full-width at half-maximum (FWHM) of 10 mm, and (6) removing slices of no signal to match the matrix size of the used templates. Note that the spatial normalization step is used only for classification purposes and that each patient's ICA maps are available in their original “patient space.” The preprocessing script is publicly available from https://dl.dropboxusercontent.com/u/33755383/algorithms_scripts.7z. A flowchart of the steps of the entire method from preprocessing to classification is shown in Figure 2.

### Independent component analysis

After initial processing, each subject's (and patient's) resting scan was input into ICA open source software (GIFT toolbox, http://icatb.sourceforge.net/groupica.htm, version: v3.0a) identical to that which generated the RSN IC templates (see *Spatial Correlation Classifier* section below). For a detailed description of ICA in fMRI see (McKeown et al., 1998). Data were decomposed into independent components using individual, spatial ICA (Infomax algorithm). An intermediate model order (number of components = 28) was chosen to achieve a balance between robustness of component spatial maps and the number of components extracted (Jafri et al., 2008; Smith et al., 2009; Biswal et al., 2010; Allen et al., 2011; Damoiseaux et al., 2012). Components for each patient for the auditory, visual, default-mode, sensorimotor, and executive control networks were visually identified by two expert viewers. The resulting component maps and associated labels were used in the machine learning analysis as the testing and training sets. A point to be aware of is that this method used network-level labeling like Allen et al. (2011) and not component-level identification like Demertzi et al. (2014).

### Machine learning

As a first attempt, and as a baseline reference, automated IC map network identification was investigated with a simple correlation classifier by calculating spatial correlation to a template (Greicius et al., 2004; Damoiseaux et al., 2008; Greicius, 2008).

More advanced classifiers included standard machine learning multi-class algorithms for the task of classifying individual components as auditory, visual, default-mode, or sensorimotor network components. These were: decision trees, perceptrons (one layer neural networks), naïve Bayes classifiers, and support vector machines (SVMs; included as a baseline machine learning classifier, see Supplementary Material). The above classifiers provide complementary methods of managing the classification task. A decision tree dichotomizes feature space by splitting at specific feature values, a perceptron finds separating hyperplanes in feature space, and the naïve Bayes classifier is a probabilistic approach that estimates conditional probability distributions. The classifiers are described in more detail below, in their respective sections and in the Supplementary Material. These algorithms are chosen because they produce interpretable models and are able to classify more than two labels at a time (multi-class classification). The decision tree and SVM algorithms used were from the open source Spider Machine Learning library freely available from http://people.kyb.tuebingen.mpg.de/spider/. The perceptron and naïve Bayes algorithms were coded by the authors and are publicly available from https://dl.dropboxusercontent.com/u/33755383/algorithms_scripts.7z. The metric used for measuring classification performance was a matching percentage (accuracy) to an expert viewer's manual network identification. The results of two expert viewers were averaged for the final accuracy.

#### Feature space

The input to the correlation, decision tree, perceptron, and naïve Bayes classifiers was a full brain *t*-statistics map of the components output from GIFT ICA software. The input feature vector is one of *t*-statistics with dimension equal to the number of voxels in the three-dimensional field of view of the brain image. A dimension reduction and tuning step was performed (excluding the spatial correlation classifier) to select a resizing parameter *R* of the three-dimensional image (i.e., from the original 53 × 63 × 46 matrix size to *R* × *R* × *R* by averaging neighboring voxel intensities, see the *Feature Dimension Reduction* section below).

#### Feature dimension reduction

Due to the high dimensionality of resting state brain volume data and IC map spatial information distribution, this method sought to improve the feature representation of the IC network map examples by resizing the image volume matrix.

Prior to running ICA, the resting data was smoothed with a 10 mm FWHM Gaussian kernel (standard in resting state analysis). This smoothing acted like a low pass spatial filter, retaining the low spatial frequency information but suppressing the high spatial frequency information. In Figure S1, it is seen that most of the information in the original component image is located in the low frequency range of *k*-space. Since an accurate approximation of the original can be made by using only the low frequency range, a smaller matrix size (or larger voxels) can be equivalently used to adequately represent the image.

The initial matrix size was 53 × 63 × 46, which contained 153,594 voxels (features). The parameter *R* was tuned in the algorithms by varying matrix sizes from 5 × 5 × 5 to 20 × 20 × 20, which reduced the number of features to 125 at the minimum and 8000 at the maximum (see Figure S1).

#### Spatial correlation classifier

Each component output from ICA was spatially correlated (Pearson correlation coefficient) with ICs of the templates which were grouped into auditory, visual, default-mode, and sensorimotor RSNs from a previous study (Allen et al., 2011). A ranking by correlation was used for the classification step (coded in MATLAB 2013a, The MathWorks, Inc., Natick, Massachusetts, United States): the template component which had the highest spatial correlation to the input subject component was chosen and its network was inherited as the predicted network label.

#### Perceptron

A perceptron is a one layer neural network that is simple in structure and computation and robust to noise present in data. It takes in a real-valued vector as input (e.g., *t*-statistics map), calculates a linear combination of the vector components (by multiplication with learned weights) and outputs 1 or -1 if the result is above or below a threshold. In this work's case, a set of four weight vectors was learned, each corresponding to a RSN label (auditory, visual, default-mode, sensorimotor, or executive control). Classification was done by taking the maximum of the following dot products: <input vector, weights of auditory class>, <input vector, weights of visual class>, <input vector, weights of default-mode class>, <input vector, weights of sensorimotor class>, and <input vector, weights of executive control class>. The learning rule chosen to update weights was the standard stochastic gradient descent (weights are updated when the error of the current example in the iteration is calculated, not when the error over all examples is calculated). The reader is directed to the work by Mitchell (1997) for a detailed description of the algorithm.

#### Naïve Bayes

Bayesian learning methods provide a probabilistic approach to inference in machine classification. The naïve Bayes classifier explicitly computes probabilities for hypotheses and is one of the most practical algorithms for various types of problems. Michie et al. (1994) provide a detailed analysis of the naïve Bayes classifier, compare it to other algorithms (decision trees, neural networks) and show that it performs as good as and in some cases better than the others. Naïve Bayes learning involves estimating conditional probabilities from the training data features (attributes) and classes (labels). The estimates are calculated by counting the frequency of feature and class combinations. Classification is done by applying Bayes rule (using the derived probability estimates) to an input vector's feature values, with the assumption that each feature is conditionally independent given the class.

Since learning with the naïve Bayes classifier is best done on discrete valued features, inputs were transformed from a continuous to a discrete range (integers 1…11) before use in the algorithm. A *Z*-transformation was applied to each whole brain component intensity map and the values were binned into 11 *Z*-score intervals: (−∞,−1.2], [−1.2, −0.9], [−0.9, −0.6], [−0.6, −0.3], [−0.3, 0], [0, 0.3], [0.3, 0.6] [0.6, 0.9], [0.9, 1.2], [1.2, 1.5], [1.5, ∞). As a result of the binning the possible values that a feature can have were reduced to the integers 1 through 11, and learning by frequency counting was made tractable. Eleven intervals were chosen to intermediately partition the *Z*-score range which had most of its values between −1.2 and 1.5.

#### Training, tuning, and testing sets

The tuning set for the above three standard machine learning classifiers (as well as for the SVM) was a randomly chosen set of 15 subjects out of the original 30 for the healthy dataset and a randomly chosen set of 12 patients out of the original 23 for the epilepsy dataset. Training and testing (and tuning) were done using leave-one-out cross-validation (LOOCV) to best estimate the model accuracy (Hastie et al., 2001) on a future, not-seen-before subject or patient who is required to be from the same population distribution as was sampled by the training set. This training and testing approach simulates a clinical scenario, where all known epilepsy patients are used to train a classifier model and one unknown patient is predicted using the model.

**Figure 3:**
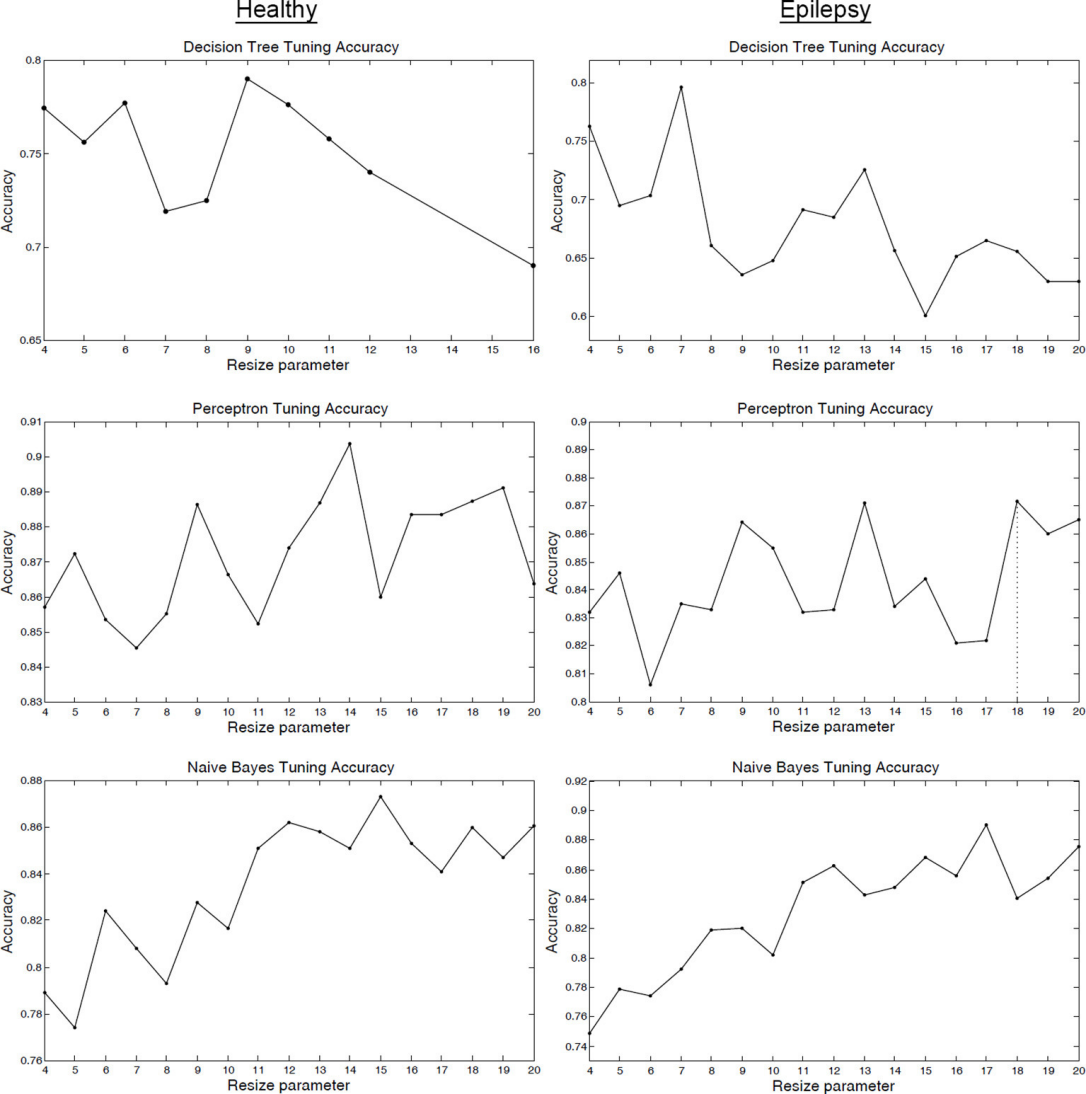
**Tuning set classifier accuracy**. Tuning set classifier accuracy as a function of the resizing parameter *R* for healthy subjects (column 1) and epilepsy patients (column 2). The value of *R* that maximized tuning set accuracy was selected for LOOCV training and testing.

Figure 3 (and Figure S4) shows how the tuning parameter was selected on the tuning sets. Usually it is unknown how the value of the parameter will affect performance for different classifiers—this is specific to the data and type of algorithm. Standard practice is to pick the parameter that maximizes a classifier's tuning set accuracy. Figure 3's accuracy function may change if a different number of components are used, however, the tuning procedure of Figure 3 will remain the same. The purpose of the tuning step is to select the optimum classifier parameter after training on a tuning or validation set. The decision to select a particular model for ICA order comes from the domain experts.

## Results

Functional network component extraction with ICA revealed distinct, consistent spatial maps of known networks for healthy subjects and epilepsy patients (see Figures S2, S3). Each healthy subject and patient possessed approximately 8 IC maps belonging to the five networks examined.

The classification method achieved high performance accuracy: IC maps derived from rs-fMRI data of epilepsy patients were classified into five different functional networks with an accuracy of 88% (average of two viewer labels, *p* < 0.001, binomial test; Table 2, Tables S1, S2). The *p*-values were calculated to be the probability that the classification accuracy would result if the classifier was guessing RSN labels at random (1/5 chance; Pereira et al., 2009), using a binomial distribution. For comparison, a Naïve Bayes classifier without tuning was trained using the full resolution of 53 × 63 × 46 and the accuracy was 86%, which was comparable to the tuned classifier but less accurate. This similar performance is reasonable for the Naïve Bayes classifier when considering the stable region in tuning accuracy for the higher resolutions as shown in Figure 3 (note that the accuracy of the decision tree classifier quickly degraded as the resolution was increased).

**Table 2:** **Accuracies of the four classifiers used on the epilepsy patient dataset**.

**Algorithm**	**Accuracy (average of Viewer1 and 2)**
	**Epilepsy (%)**
Correlation classifier	69
Decision tree	70
Perceptron	81
Naïve bayes	88

*Note that for the correlation classifier the executive control network was not included*.

The focus of the study was the epilepsy dataset—the healthy dataset was used to gain some context for which to compare the epilepsy classifier performance and to inspect IC map consistency between the groups. A perceptron classifier was able to classify healthy subject IC maps with 90% accuracy (Table S1; *p* < 0.001, binomial test).

Some network components, if spatially dissimilar enough from others, were classified with high accuracy as was seen with the visual network (100% for epilepsy) for both healthy subjects and epilepsy patients (Table 3, Table S3). Tables S4, S5 list the sensitivity, specificity, and positive and negative predictive values for the perceptron classifier (healthy) and naïve Bayes classifier (epilepsy). Additionally, a separate analysis using only 18 temporal lobe epilepsy patients was carried out to verify the method's validity and performance on a homogeneous epilepsy set (see Table S6). Patient 19 who had no EEG correlation was excluded. The accuracy was 86.2%, which closely matched the 23 patient accuracy of 88%. This consistent result using the homogeneous group gives strong evidence supporting the performance of the classification method.

**Table 3:** **Confusion matrices for the naïve Bayes classifier on epilepsy patients' components for Viewer 1 and 2**.

	**Predicted auditory**	**Predicted visual**	**Predicted default-mode**	**Predicted motor**	**Predicted executive control**	**Accuracy (%)**
**CONFUSION MATRIX FOR VIEWER 1 LABELS**						
Auditory	34	0	1	3	0	89.5
Visual	0	43	0	0	0	100
Default-mode	2	0	36	3	0	87.8
Motor	7	1	8	44	0	73.3
Executive control	0	0	0	3	15	83.3
**CONFUSION MATRIX FOR VIEWER 2 LABELS**						
Auditory	24	0	0	2	0	92.3
Visual	0	41	0	0	0	100
Default-mode	1	2	36	1	0	90
Motor	6	0	3	47	0	83.9
Executive control	0	0	0	3	15	83.3

*Note that accuracy is defined for each class (network) as a matching rate to the viewer identified labels*.

Relatively simple, but standard, machine learning algorithms proved successful in this clinically oriented multi-class classification task (Table 2). The method's performance and utility in clinical workflow are discussed in more detail in the discussion section.

**Figure 4:**
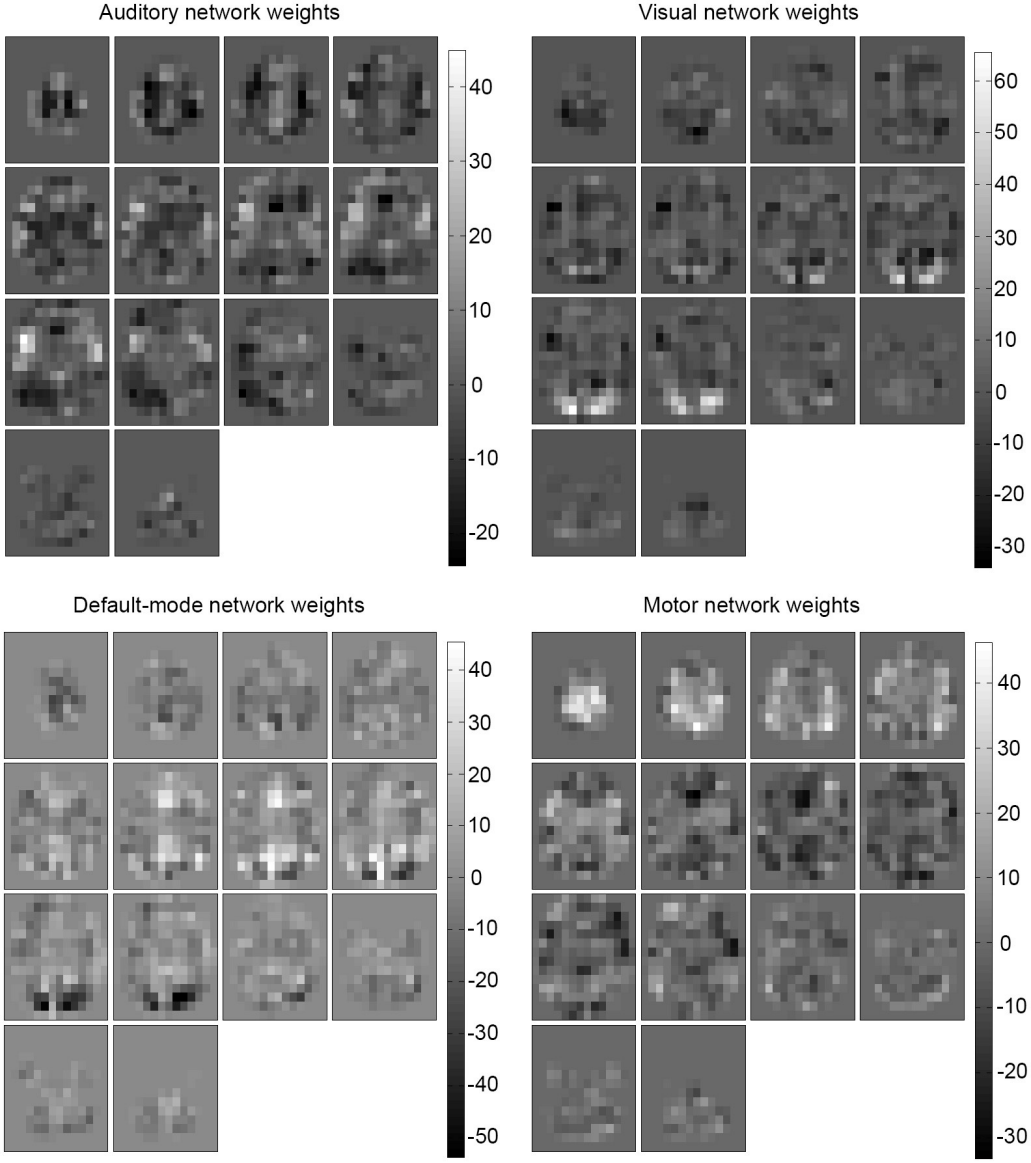
**Learned weights for the perceptron**. Learned weights for the perceptron classifier (healthy) are shown for each predicted network (executive control not shown). This reveals that the most influential areas correspond anatomically to their respective network location.

**Figure 5:**
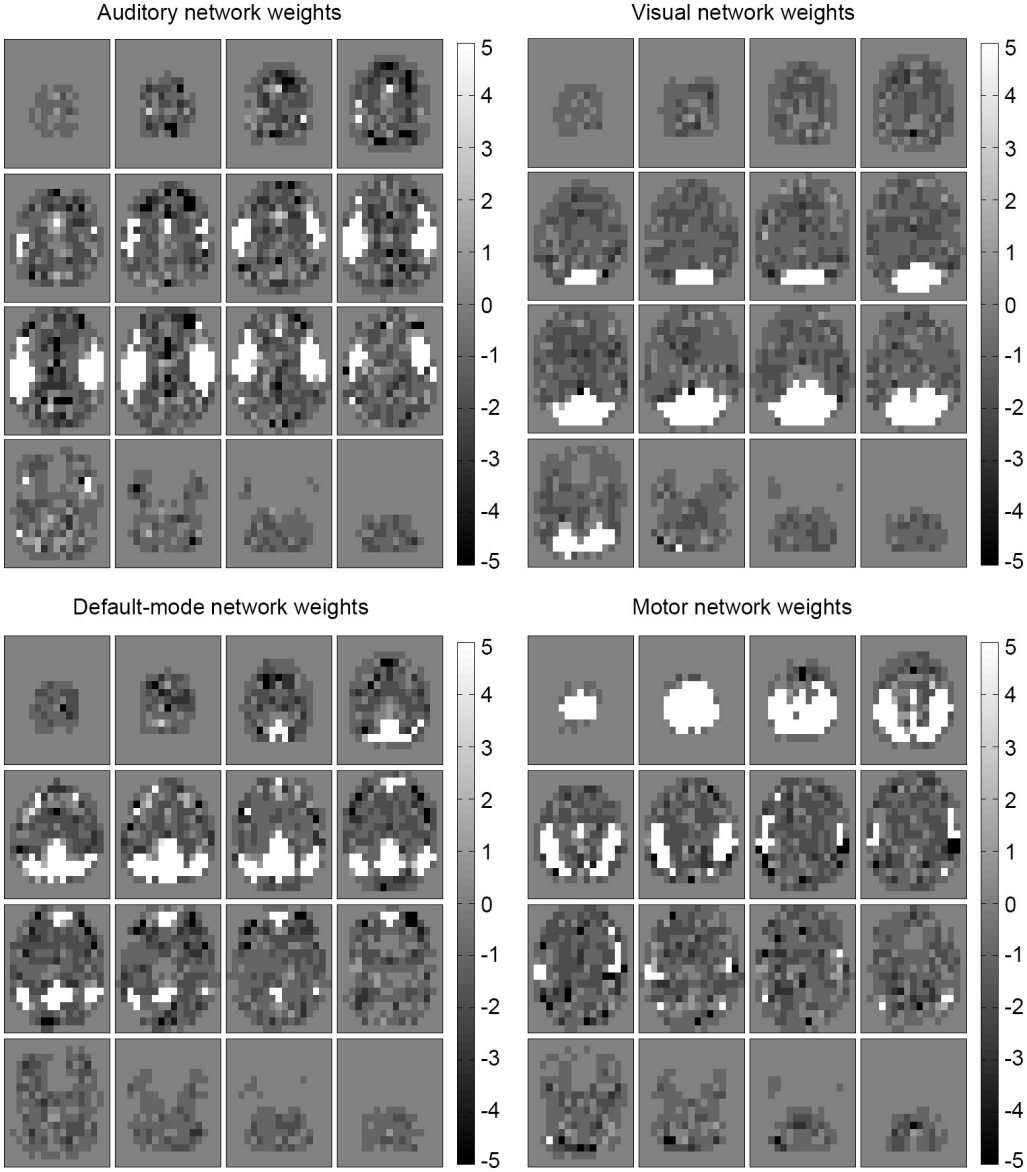
**Influential features and areas for naïve Bayes**. Most likely values (learned conditional probabilities) for the naïve Bayes classifier (epilepsy) are shown for each predicted network (executive control not shown). The most influential areas correspond anatomically to their respective network location. Note that the values were shifted to be centered at zero (from the original [1,11] range) to match the perceptron weight appearance.

Examining the weights of the perceptron and the conditional probability estimates of the naive Bayes classifier revealed that the concept learned by the algorithms was an anatomical, spatial representation of the four networks. The weights that most influenced classification were located at the functional and anatomical regions of each of the four (executive control not shown) respective networks (Figures 4, 5).

The benefits of automating this mapping and labeling task were clearly seen when the run time of the algorithms was considered. Once trained, they were able to classify a single subject's components in a tenth of a second (on an Intel Core 2 CPU, 3 Ghz, 8 GB RAM), much less than the time required (30+ min) for manual examination and labeling where nearly 30 ICA volumes are examined slice by slice.

## Discussion

The purpose of this work was to develop a RSN labeling method which has utility in clinical rs-fMRI mapping. The method provides a fast way to extract, label, and organize IC maps. This offers value in the clinical workflow by reducing the time consuming task done by researchers or clinicians of applying ICA and searching through many unordered IC maps for components belonging to relevant networks. The output of the method was an ordered list of IC maps with network-level labels (Figure 1), which the clinician can use as an organizational tool that provides complementary functional network information and probable network identification. It is important to note that the method's brief interpretation of the maps must still be clinically verified in the workflow. The study by Mitchell et al. (2013) provided evidence about the specific clinical validity of rs-fMRI and machine learning derived network maps, showing that the maps corresponded to cortical stimulation mapping of the motor and language networks. The motivation for this article's work was to incorporate the RSN classification method to complement and reinforce existing procedures as analyzed by Zhang et al. (2009). Whereas, the study by Mitchell et al. (2013) trained their classifier on a set of normal subjects and then tested on a set of six epilepsy patients, this study both trained and tested the classifiers on epilepsy patients which provided more consistency in the method and more meaningful results of accuracy with respect to the viewer labels. Using a healthy subject classifier for epilepsy patients would not be clinically acceptable (see below).

In resection treatment for epilepsy, vascular lesions and tumors it is important to preserve healthy functional tissue. Presurgical mapping involves identifying healthy functional tissue that should be preserved during resection. Damage to the default-mode network, for example, may affect cognitive functions such as planning for the future, navigation of social interactions, and memory retrieval (Buckner et al., 2008) and damage to the motor network may lead to motor deficits. Functional connectivity analysis of rs-fMRI in neurosurgery has shown promise as a tool for diagnosis and surgical planning when used preoperatively to localize areas of eloquent cortex, to provide prognostic information by suggesting the degree of morbidity resulting from removal of specific areas of brain tissue, and to inform the surgeon of safe maximal resection boundaries (Lang et al., 2014).

The naïve comparison of the healthy normal (90%) and epilepsy classifier accuracy (88%) suggests that a high level of accuracy can be achieved with both populations. Note that a group comparison was not performed and the group difference was not rigorously tested. The near 90% accuracy for the patient sample is excellent considering that the classification problem itself is not binary but multi-class (5-class in this case, with a random guessing chance of ≈20%). These results support consequent work with a more diverse patient sample (different epilepsy types, seizure burdens, and anatomy) and more functional network coverage to better represent the true clinical epilepsy population and improve clinical relevance.

**Figure 6:**
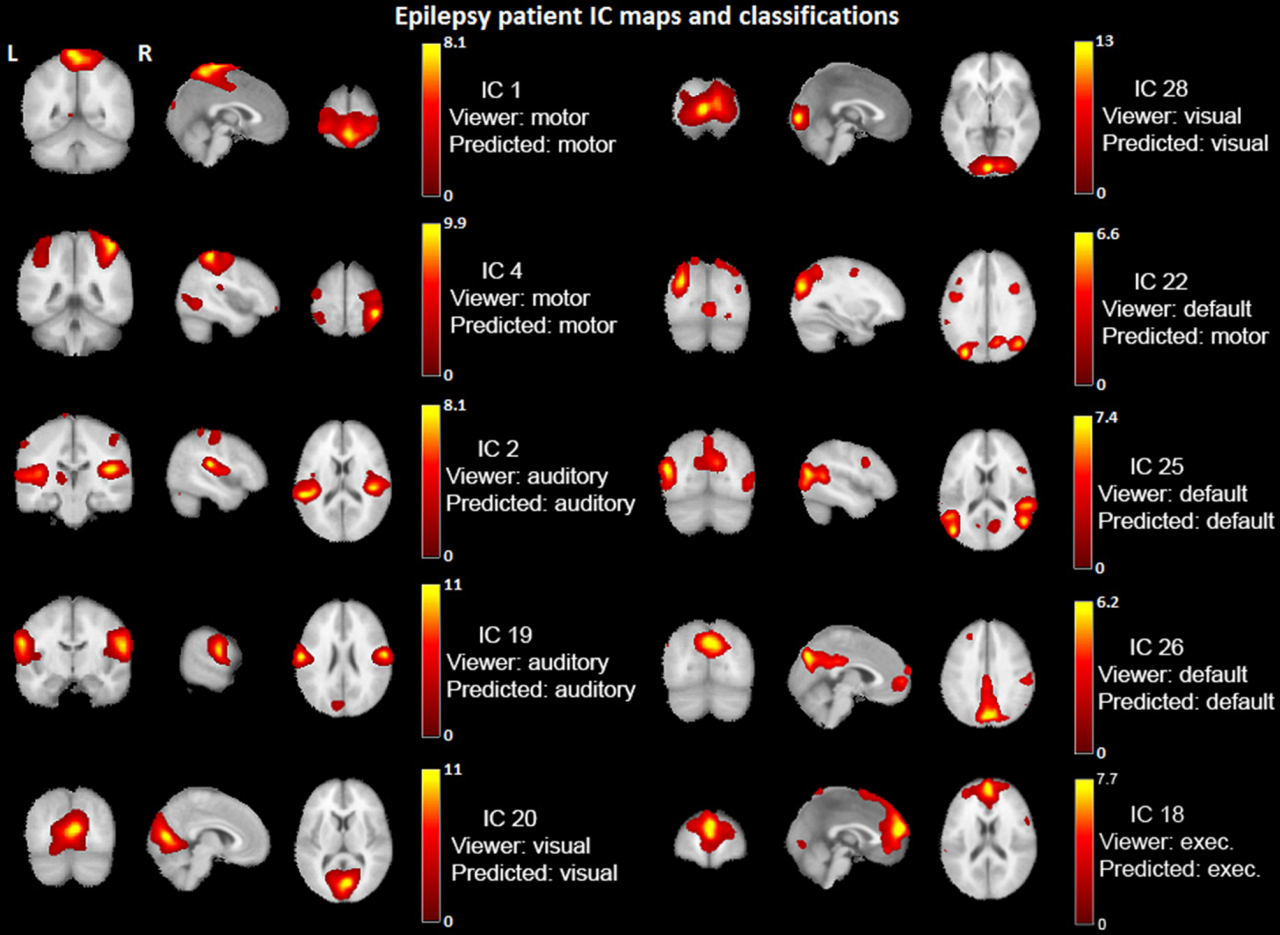
**Example epilepsy patient's IC maps classification**. IC spatial maps (*t*-statistics > 2.0) identified into networks by the viewers and classified by the Naïve Bayes algorithm. IC #22 is the only one misclassified for this patient. The underlay is a standard MNI_avg152T1 AFNI template.

Figure 6 shows an example patient's IC maps labeled by the classifier, with only a single (out of ten) misclassified component with regard to the expert viewers. It is motivating to see such high accuracy results for a multi-class classifier on a dataset of 23 patients. Mathematically and statistically speaking, the performance will improve as the amount of training data increases—the classifier will be able to learn a better model representation of the task. Also, improvement of the functional accuracy or appropriateness of the provided, labeled RSNs (e.g., higher model order for more spatial detail) will improve the clinical utility of the method.

The accuracy for future patient maps, or the true risk on the population, depends on how well the training set represents the clinical population. The main statistical principle behind the applicability of the method to clinical populations is the use of a training sample that is independently and identically distributed (i.i.d.) and drawn from the clinical population distribution. With such a training sample, the classifier can learn the task and use it for a new patient also from the same clinical population and drawn i.i.d. from it (see Appendix in the Supplementary Material Text; Shalev-Shwartz and Ben-David, 2014). Because of this, using a healthy subject classifier for epilepsy patients would not acceptable in machine learning and clinically. This work followed the standard method for regression and classification and for the purpose of showing feasibility, only considered a limited epilepsy population sample.

A desirable property of this analysis is that no assumptions, constraint, or domain knowledge has to be specified beforehand about the clinical population distribution (one does not need to require that epilepsy patient networks are inherently different from healthy subjects, and also does not need to require that they are inherently similar). This is because the training sample set encodes the population distribution—the functional network information is contained in the population sample itself. The machine learning algorithm learns the pattern of the spatial maps from the distribution (Figures 4, 5) and can use it for future, new patients from the same distribution.

The naïve Bayes probability values and perceptron weights calculated using the trained ICA model (Figures 4, 5) and can be used to suggest the level of confidence of a classification. A map that is distributed as expected (in the population) should have only one large probability value (the classification step is done by taking the label with a maximum probability). The presence of several large, similar probabilities can reveal that a patient's ICA map spans several learned networks and can suggest a lower level of confidence for the classification. These probability values will be available along with the output label by the classifier as an indicator of the confidence of the result.

The healthy and epilepsy IC spatial maps showed clear network differentiation and were comparable to maps of previous intermediate model order ICA studies (Beckmann et al., 2005; Damoiseaux et al., 2006; Calhoun et al., 2008; Smith et al., 2009; Biswal et al., 2010; Allen et al., 2011). IC maps for each subject consisted of artifactual components, functional networks, and sometimes a superposition of networks. Approximately 8 components per subject were identified to be signal from the auditory, visual, default-mode, sensorimotor and executive control networks (Figures S2, S3, executive control not shown). The original data contains all of the acquired resting scan functional information and all extracted components in “patient space” will be available to the clinician to view when examining the rs-fMRI scan, along with the set of the labeled components.

An intermediate model order was chosen for the purposes of extracting robust spatial maps and to be consistent with the ICA studies in the literature (see *ICA* section above). This ICA model order has been shown to closely correspond to task fMRI activation maps by Smith et al. (2009), who investigated a model order of 20 (as well as 70). In general, if a higher model order is used then some networks may be split into subcomponents (Smith et al., 2009). A high model order is more clinically demanding and requires that many more maps be inspected but can offer finer detail of relevant network maps (Zhang et al., 2009).

For showing feasibility of the method, the auditory, visual, default-mode, sensorimotor, and executive control networks were used (this excluded the fronto-parietal and cerebellar networks as in Smith et al., 2009). Note that the visual, sensorimotor and default-mode networks in this study contained multiple ICs. For this organization, the authors used the terminology analogous to that of ROIs being grouped into different functional networks (e.g., ROIs as in Dosenbach et al., 2010).

A limitation of this work is that a complete functional network coverage of patients was not used. This study did not directly use a language network component map. A complementary language network IC map that covers the frontal lobe regions (see Smith et al., 2009), Figure 1, IC 10) can be a valuable addition to the ICA extraction and classification method and would improve clinical relevance. Additional networks to include are the salience and cerebellum networks covered by fronto-parietal maps IC 9 and 10 and IC 5, respectively, in Figure 1 of Smith et al. (2009). To develop this method for clinical-grade performance, a complete RSN coverage is required.

The method and its performance are discussed below in more detail. It is seen from the confusion matrix (Table S3) that for healthy subjects the perceptron classifier had a tendency to misclassify the auditory components to be sensorimotor components and vice-versa (e.g., subject row 2 IC15 and subject row 4 IC3 in Figure S2). Similarly, the perceptron mismatched mostly the visual and default-mode network components (e.g., subject row 4 IC5 and subject row 5 IC22 in Figure S2). This is understandable since these pairs of networks had some components that had spatial extents very near one another and visually appeared similar to a human viewer.

For epilepsy patients the naïve Bayes classifier showed the same tendency for the auditory-sensorimotor pair (e.g., subject row 1 IC23 and subject row 5 IC10 in Figure S3). An interesting note is that the visual network components were perfectly classified for epilepsy patients (Table 3).

Advantages of the machine learning method in this study over the Demertzi et al. (2014) component matching method are the model tuning and validation (which improves accuracy) as well as the multivariate capabilities. Another advantageous property of the chosen machine learning classifiers is that the learned concept models are readily interpretable. For the perceptron, features with high weight values are more influential to classification and for the Bayesian classifier, most probable feature values influence classification toward the respective class. As seen in Figures 4, 5, the two classifiers learned the spatial, anatomical IC map network location for the four networks. The classifiers learned the RSN patterns of the sampled patients well and were then able to classify networks accurately using the learned model. Note that a group comparison (healthy vs. epilepsy) of the classifiers was not performed and the IC maps and resulting classifier model weights were inspected between groups to provide some insight into the consistency of networks.

## Conclusion

Research studies are providing increasing evidence of the value rs-fMRI offers to neurosurgery through functional mapping (Lee et al., 2013; Lang et al., 2014). Interpretation and labeling of RSNs provides important information about a patient's functional status, with advantages over task fMRI mapping. Machine learning classification, trained on the gold standard of a clinician's labeling, has potential to aid the clinical workflow and reduce the time demand faced by clinicians in manually inspecting many ICA maps.

This work showed the excellent performance of ICA network classification for healthy subjects and epilepsy patients and proposed a clinical use for aiding investigation and evaluation of functional networks done during presurgical mapping. Consistent and reliable automation is a desirable addition that can reduce investigator bias inherent in visual labeling. The authors believe that neuroimaging interpreters can benefit from complementary machine learning methods which provide automatic labeling and organization of rs-fMRI maps. This clinician and machine learning combination takes advantage of the strengths of both humans and computers: human expert interpretation and high-level cognition and the computer's ability to quickly and reliably handle many variable calculations.

This work provided evidence of a successful single patient RSN labeling method and with a larger, more diverse training sample, expanded functional network coverage, and incorporation of new classifier features (demographics, clinical variables, anatomical image features) its performance and clinical utility can be improved.

## Author contributions

SV performed the majority of the analysis and wrote the manuscript. WG, VN, and JS performed analysis and assisted in writing and editing of the manuscript. RB, AA, MM, JR, ED, and VP assisted in writing and editing of the manuscript and provided guidance for the study.

## Funding

This project was supported by awards from the National Institutes of Health (http://www.nih.gov; R41NS081926 to ED, JR, VP; RC1MH090912 to MM, VP; K23NS086852 to VP; ICTR KL2 as part of UL1TR000427 to VP; and T32EB011434 to MM, SV). Support was also provided by the Foundation of ASNR's Comparative Effectiveness Research Award (http://www.asnr.org/foundation/awards/award_cer.shtml) to VP.

### Conflict of interest statement

The authors declare that the research was conducted in the absence of any commercial or financial relationships that could be construed as a potential conflict of interest.
