# Supplementary material for: Classification and Extraction of Resting State Networks Using Healthy and Epilepsy fMRI Data
Source: Front Neurosci. 2016 Sep 27;10:440. doi: 10.3389/fnins.2016.00440 (PMC5037187; doi:10.3389/fnins.2016.00440)
Supplement: Supplementary file 1 [file DataSheet1.DOCX]

***Supplementary Material***

**Classification and Extraction of Resting State Networks Using Healthy and Epilepsy fMRI Data**

**Svyatoslav Vergun*, Wolfgang Gaggl, Veena A. Nair, Joshua I. Suhonen, Rasmus M. Birn, Azam S. Ahmed, M. Elizabeth Meyerand, James Reuss, Edgar A. DeYoe, Vivek Prabhakaran**

*** Correspondence:** Svyatoslav Vergun: svergun@wisc.edu

**1 Supplementary text**

**1.1 Methods**

**1.1.1 Decision tree (C4.5)**

The decision tree is a supervised classifier that has proved to be very practical and successful in machine learning. It is a method of approximating discrete valued target functions (e.g. one of four network labels) in which the learned function is represented by a decision tree (Mitchell, 1997). Here we used the C4.5 decision tree algorithm developed by Quinlan (1993) that selects features at each node in the tree structure by using the information gain measure. The algorithm has a preference for small trees over large ones. The reader is directed to the work by Quinlan (1993) and Mitchell (1997) for a detailed description of the algorithm.

**1.1.2 Support vector machine**

Support vector machines (SVMs) are one of the most popular algorithms for learning because they have the advantages of flexibility in representing complex functions through use of the kernel trick and resistance to overfitting through retaining a fraction of the training examples (Russell and Norvig, 2010). They were included in this analysis as a reference classifier for comparison to the other methods. SVMs construct a maximum margin separator, a discrimination (decision) boundary, with the largest possible distance away from example points which generalizes well for future, unseen data. The algorithm finds a hyperplane that discriminates between two classes in high-dimensional space by solving a convex optimization problem. Usually data are not linearly separable and a soft margin is used that allows some examples to be misclassified (Cortes and Vapnik, 1995). The parameter C controls the amount of penalty assigned to misclassified points.

The software default value of C = ∞ was used in this study, where no misclassification is allowed (hard-margin case). We also tested the classifier with C = 1, allowing misclassification (soft-margin case), and the resulting performance was comparable to and only slightly better than the default case. SVM classification was carried out using the Spider Machine Learning environment (Weston et al., 2005) as well as custom scripts run in MATLAB. Multi-class classification was performed with an algorithm developed by Weston and Watkins (1998) that considers all data at once and solves a single optimization problem.

**1.2 Discussion**

The feature reduction step reduced redundancy in the spatial maps—resizing the matrix equivalently removed the unnecessary higher spatial frequency information in the image, which is appropriate since the IC spatial maps exhibited low frequency patterns (see S1 Fig). Note that coarse resolution (≈15×15×15) is only used for labeling.

SVM tuning accuracy was the most stable over our tuning parameter range (S4 Fig), showing that the SVM algorithm was more independent of the parameter value than the other classifiers.

The initial, reference spatial correlation classifier, although statistically significant, was greatly outperformed by the standard machine learning classifiers. The correlation classifier, in a high-level view, averaged all of the features into a single measure (the correlation value) and this is a great oversimplification of the problem. This kind of under-fitting of the problem leads to a very limited representation of the model. The standard machine learning classifiers used much more of the image features allowing for richer model representations. The perceptron and naïve Bayes algorithms performed comparably well for both the normal subjects and patients and were the most successful. Even though these are relatively simple machine learning algorithms, they proved to be very effective.

The naïve Bayes classifier outperformed the linear separators (perceptron and SVM) for epilepsy patients. Its accuracy remained high and consistent for normal and epilepsy data, whereas the perceptron’s (and SVM’s) accuracy decreased from normal subjects to epilepsy patients. An advantage of the Bayesian classifier is that it can handle inseparable data better than the hard linear boundary classifiers since it constructs probability distributions over the data space. Using a soft-margin SVM can solve the problem of inseparable data but this requires tuning of an extra parameter (the misclassification C parameter). Simpler model classifiers do not have as many hyperparameters as more advanced ones (e.g. soft-margin SVM: C parameter; multi-level neural networks: number of layers, number of hidden nodes) which removes a complication of the need to tune extra parameters but may come with a cost to potential performance.

It is seen that the decision tree tuning accuracy decreased with increasing ICA map resolution (R > 9), while the accuracy for the other classifiers tended to increase. This may be due to over-fitting of the problem by the decision tree and increasing the dimension of input examples did not add performance value.

The representation of RSNs with ICA t-statistic spatial maps proved to be adequate for this application and resulted in high accuracy classification. The initial dimension of the maps was quite large, but the matrix resizing step successfully reduced it, improving classification accuracy (see Fig 3 in the main text) and computation speed.

The auditory network map used has a strong spatial correlation to the cognitive language speech domain (see Smith et al., 2009, Figures 1 and 2). This auditory IC map spatially matched activation patterns seen in the temporal lobes of the patients during language task experiments (true and false sentence discrimination, and text reading for comprehension). Also, there were signal components, excluded in the analysis, that spatially matched language activity patterns seen in the frontal lobe regions identified from the same language task experiments. This is to be cautiously interpreted, however, since no quantification of the match was investigated.

**1.3 Appendix**

Let Accuracy be defined as 1-Loss, this represents the correctness of an output label (Loss = 1 if the label does not match the expert clinician’s decision, Loss = 0 if it matches). Loss is used conventionally in the research area and it is an equivalent measure to ‘poor accuracy’.

One wants the Loss on the population distribution, using the algorithm, to be minimal: LossD(Algorithm) < epsilon, for some small number epsilon.

In practice one can only calculate the Loss on the training sample S, LossS(Alg). When the sample S (also called the training set) is identically and independently distributed (i.i.d.) according to the Distribution D, one can guarantee that LossS(Alg) is a good approximation of LossD(Alg), | LossS(Alg) - LossD(Alg)| < epsilon2, given enough training sample patients. The more training patients one has to train the algorithm, the less the true Loss will deviate from the Loss on the sample, LossS(Alg) ≈ LossD(Alg). It is expected, in the asymptotic sense, as the training set size grows sufficiently large, that the algorithm performance trained on the training set will match true performance on the population distribution.

This result from statistical machine learning theory (Shalev-Shwartz and Ben-David, 2014) gives us a statistical way to generalize the method from a sample to a population distribution (e.g. epilepsy population).

**2 References**

Cortes, C., Vapnik, V., 1995. Support-vector networks. Machine learning 20, 273-297.

Mitchell, T.M., 1997. Machine learning. 1997. Burr Ridge, IL: McGraw Hill 45.

Quinlan, J.R., 1993. C4. 5: programs for machine learning. Morgan kaufmann.

Russell, S.J., Norvig, P., 2010. Artificial intelligence : a modern approach, 3rd ed. Prentice Hall, Upper Saddle River, N.J.

Shalev-Shwartz, S., Ben-David, S., 2014. Understanding Machine Learning: From Theory to Algorithms. Cambridge University Press.

Smith, S.M., Fox, P.T., Miller, K.L., Glahn, D.C., Fox, P.M., Mackay, C.E., Filippini, N., Watkins, K.E., Toro, R., Laird, A.R., 2009. Correspondence of the brain's functional architecture during activation and rest. Proceedings of the National Academy of Sciences 106, 13040-13045.

Weston, J., Elisseeff, A., BakIr, G., Sinz, F., 2005. The spider machine learning toolbox. Resource object oriented environment. Available at: http://people.kyb.tuebingen.mpg.de/spider/main.html [accessed April 2014].

Weston, J., Watkins, C., 1998. Multi-class support vector machines. Technical Report CSD-TR-98-04. Department of Computer Science, Royal Holloway, University of London.

**3 Supplementary figures**


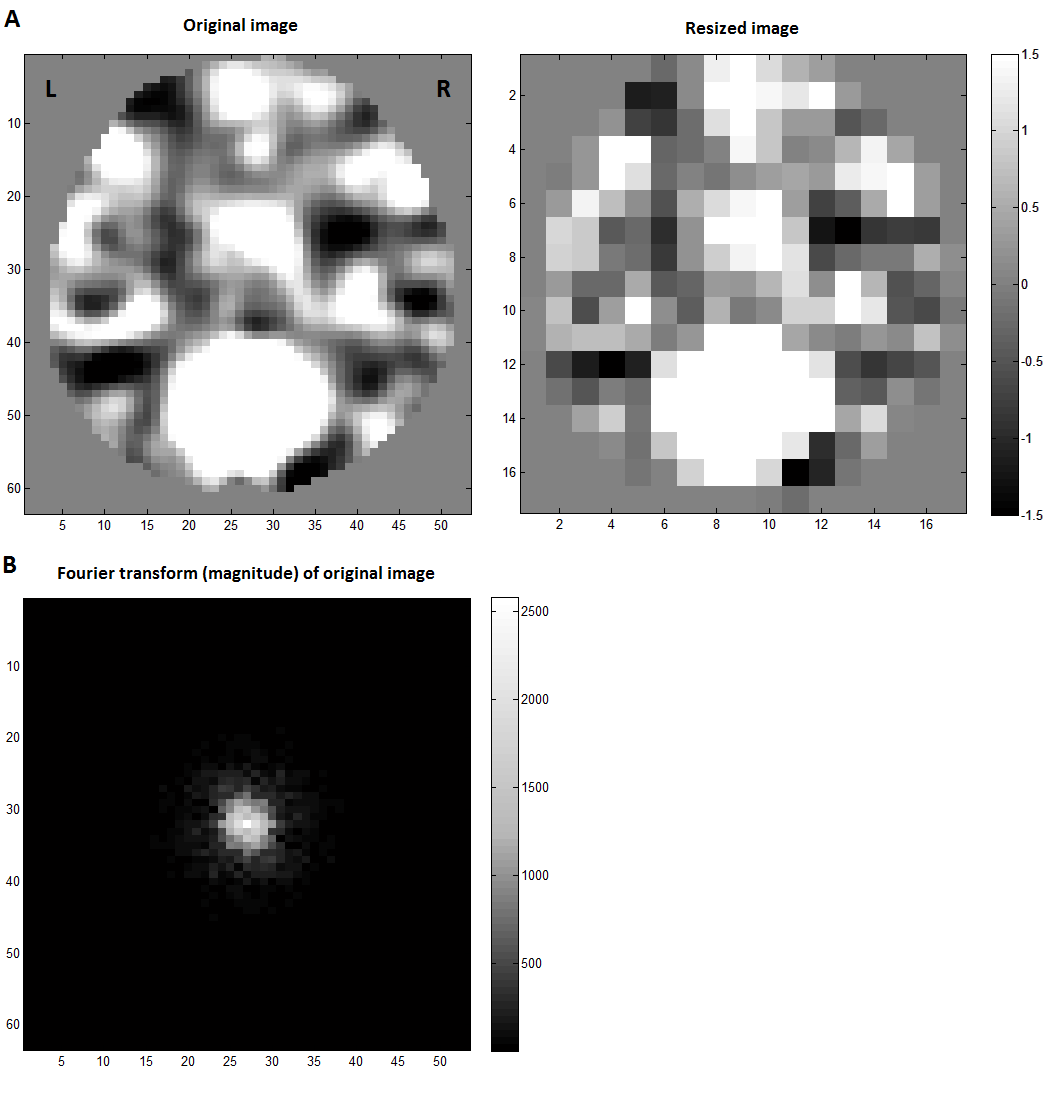


**Supplementary1 (S1) Figure**. Visual network and its spatial frequency. (A) Example visual network independent component resized from the original matrix size of 53×63×46 to 17×17×17. (B) Fourier transform of the original component (magnitude image) showing that most of the information is located in the low frequency range of k-space.


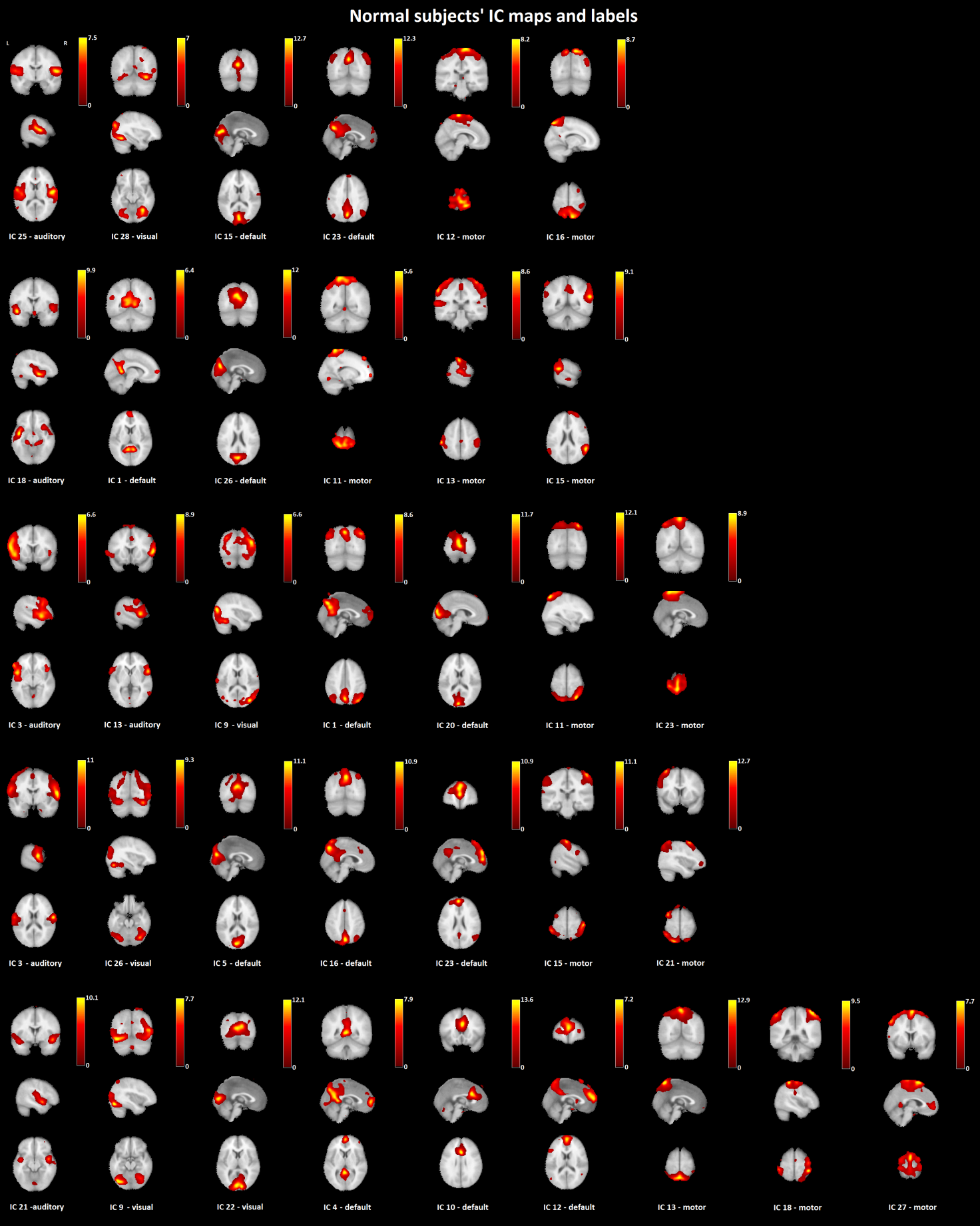


**S2 Figure**. Example healthy subjects’ components. The subjects are ordered by rows and their IC maps (t-statistics > 2.0) are in columns. The underlay is a standard MNI_avg152T1 AFNI template.


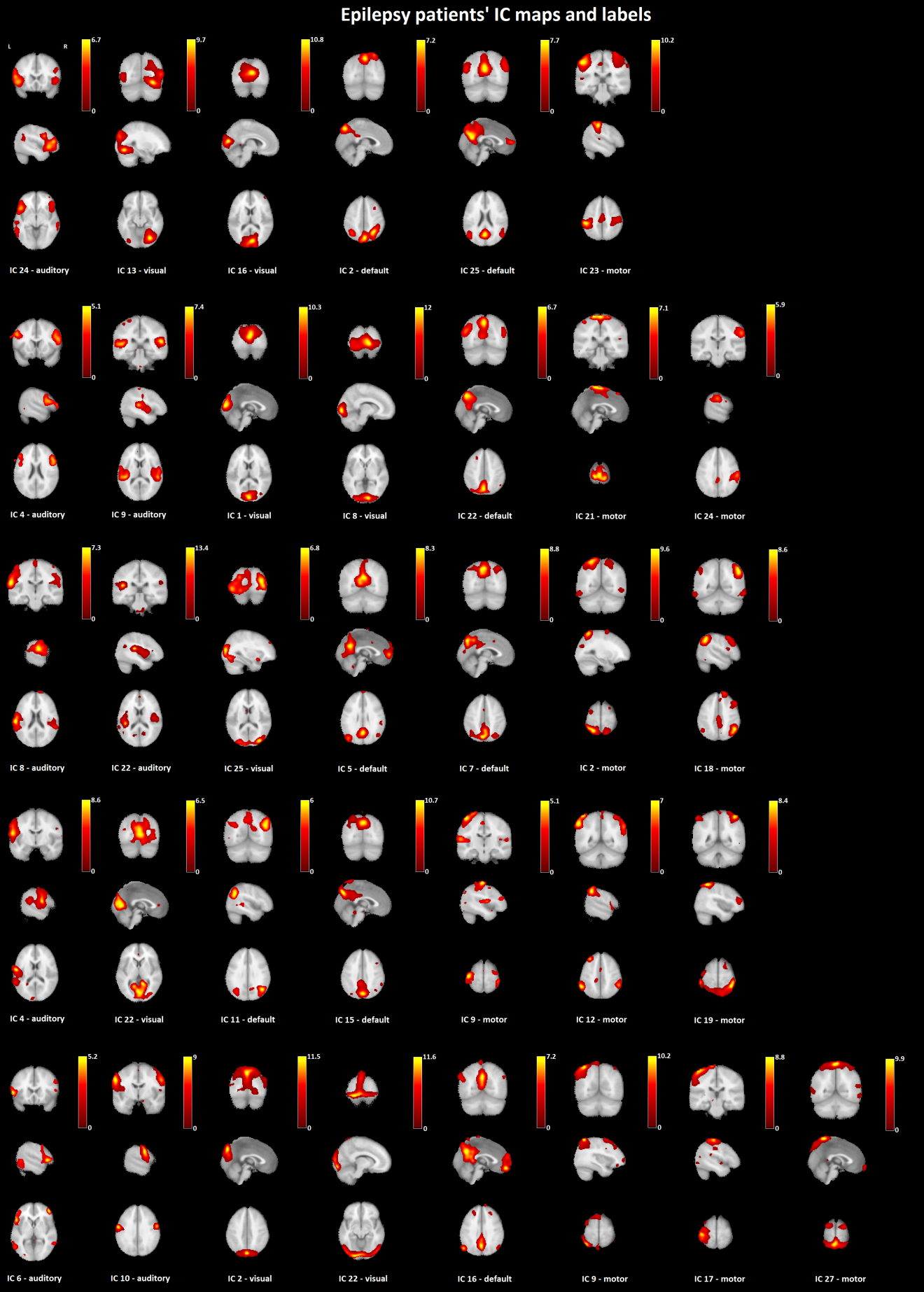


**S3 Figure**. Example epilepsy patients’ components. The patients are ordered by rows and their IC maps (t-statistics > 2.0) are in columns. The underlay is a standard MNI_avg152T1 AFNI template.


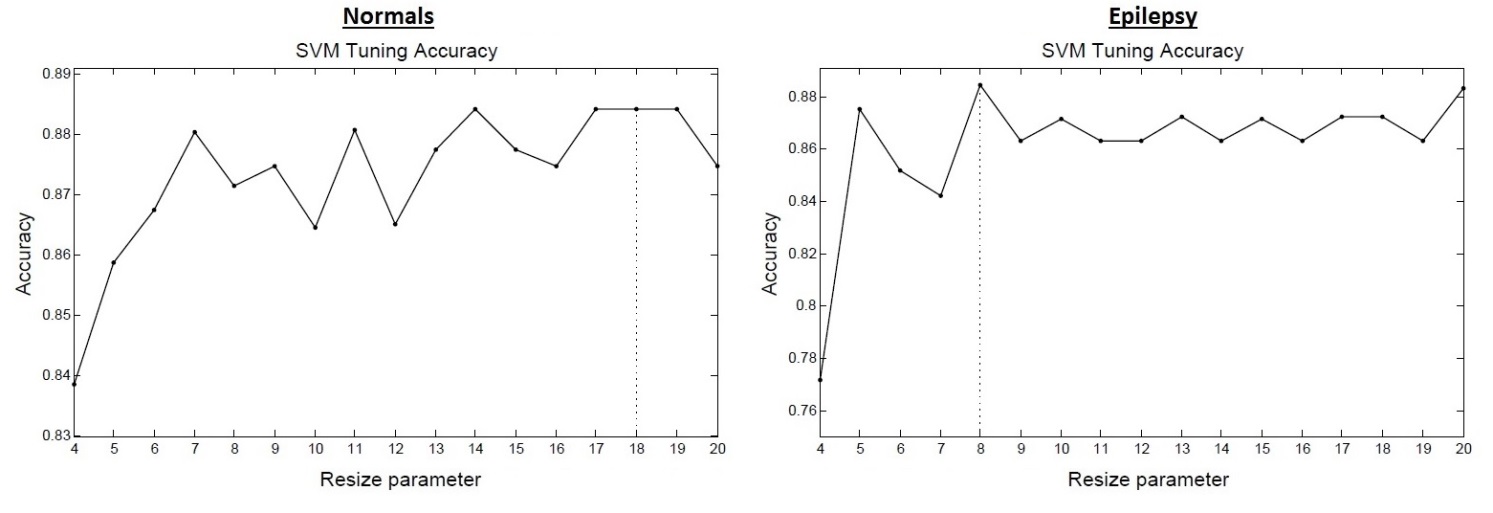


**S4 Figure**. SVM tuning accuracy. SVM tuning set classifier accuracy as a function of the resizing parameter R for healthy subjects (column 1) and epilepsy patients (column 2).

**4 Supplementary tables**

**Supplementary1 (S1) Table**. Accuracies of the classifiers on healthy normal and patient datasets.

| Algorithm | Accuracy | |
| --- | --- | --- |
|  | Healthy | Epilepsy |
| **Correlation Classifier** | 63% | 69% |
| **Decision Tree** | 80% | 70% |
| **Perceptron** | 90% | 81% |
| **Naïve Bayes** | 88% | 88% |
| **SVM** | 90% | 81% |

**S2 Table**. 23 epilepsy patient IC table and LOOCV errors for the Naïve Bayes classifier (Viewer 1). Total accuracy = 86%. 1 – motor, 2 – default-mode, 3 – visual, 4 – auditory, 5 – executive control.

| **Patient #** | **Spatial map networks (top row) and classified output labels (bottom row)** | | | | | | | | | | | | | **Error** | **Temp. focus** |
| --- | --- | --- | --- | --- | --- | --- | --- | --- | --- | --- | --- | --- | --- | --- | --- |
| patient 1 | 1 | 1 | 1 | 2 | 2 | 3 | 3 | 4 | 4 | 5 |  |  |  | 0.4 | T |
|  | 2 | 4 | 1 | 2 | 2 | 3 | 3 | 1 | 1 | 5 |  |  |  |  |  |
| patient 2 | 1 | 1 | 2 | 3 | 3 | 5 |  |  |  |  |  |  |  | 0.167 | T |
|  | 1 | 1 | 2 | 3 | 3 | 1 |  |  |  |  |  |  |  |  |  |
| patient 3 | 1 | 1 | 1 | 1 | 2 | 2 | 2 | 3 | 3 | 3 | 4 | 4 | 5 | 0 | T |
|  | 1 | 1 | 1 | 1 | 2 | 2 | 2 | 3 | 3 | 3 | 4 | 4 | 5 |  |  |
| patient 4 | 1 | 1 | 1 | 2 | 3 | 3 | 4 | 5 |  |  |  |  |  | 0 | T |
|  | 1 | 1 | 1 | 2 | 3 | 3 | 4 | 5 |  |  |  |  |  |  |  |
| patient 5 | 1 | 3 | 4 | 5 |  |  |  |  |  |  |  |  |  | 0 | - |
|  | 1 | 3 | 4 | 5 |  |  |  |  |  |  |  |  |  |  |  |
| patient 6 | 1 | 1 | 2 | 3 | 4 | 4 | 4 |  |  |  |  |  |  | 0.143 | T |
|  | 1 | 3 | 2 | 3 | 4 | 4 | 4 |  |  |  |  |  |  |  |  |
| patient 7 | 1 | 1 | 2 | 3 | 3 | 4 | 4 | 5 |  |  |  |  |  | 0.125 | T |
|  | 1 | 1 | 2 | 3 | 3 | 1 | 4 | 5 |  |  |  |  |  |  |  |
| patient 8 | 1 | 1 | 1 | 3 | 3 |  |  |  |  |  |  |  |  | 0.4 | T |
|  | 1 | 2 | 4 | 3 | 3 |  |  |  |  |  |  |  |  |  |  |
| patient 9 | 1 | 1 | 2 | 3 | 3 | 4 | 4 | 4 | 5 |  |  |  |  | 0.33 | T |
|  | 2 | 4 | 2 | 3 | 3 | 4 | 4 | 4 | 1 |  |  |  |  |  |  |
| patient 10 | 1 | 1 | 1 | 2 | 3 | 3 | 4 | 4 | 5 |  |  |  |  | 0.22 | T |
|  | 2 | 1 | 1 | 2 | 3 | 3 | 4 | 4 | 1 |  |  |  |  |  |  |
| patient 11 | 1 | 1 | 1 | 1 | 2 | 2 | 3 | 3 | 4 | 4 |  |  |  | 0.2 | T |
|  | 1 | 1 | 2 | 1 | 4 | 2 | 3 | 3 | 4 | 4 |  |  |  |  |  |
| patient 12 | 1 | 1 | 2 | 2 | 3 | 3 | 4 |  |  |  |  |  |  | 0.143 | T |
|  | 2 | 1 | 2 | 2 | 3 | 3 | 4 |  |  |  |  |  |  |  |  |
| patient 13 | 1 | 1 | 1 | 2 | 2 | 3 | 3 | 4 | 4 | 5 |  |  |  | 0 | T |
|  | 1 | 1 | 1 | 2 | 2 | 3 | 3 | 4 | 4 | 5 |  |  |  |  |  |
| patient 14 | 1 | 1 | 1 | 2 | 2 | 2 | 3 | 3 | 4 | 5 |  |  |  | 0 | T |
|  | 1 | 1 | 1 | 2 | 2 | 2 | 3 | 3 | 4 | 5 |  |  |  |  |  |
| patient 15 | 1 | 1 | 2 | 2 | 3 | 4 | 4 | 5 |  |  |  |  |  | 0.125 | - |
|  | 1 | 2 | 2 | 2 | 3 | 4 | 4 | 5 |  |  |  |  |  |  |  |
| patient 16 | 1 | 1 | 1 | 1 | 1 | 2 | 2 | 3 | 3 | 3 | 4 | 4 | 5 | 0.077 | T |
|  | 1 | 1 | 4 | 1 | 1 | 2 | 2 | 3 | 3 | 3 | 4 | 4 | 5 |  |  |
| patient 17 | 1 | 1 | 2 | 3 | 3 | 5 |  |  |  |  |  |  |  | 0 | T |
|  | 1 | 1 | 2 | 3 | 3 | 5 |  |  |  |  |  |  |  |  |  |
| patient 18 | 1 | 1 | 2 | 2 | 2 | 3 | 4 | 4 | 4 | 5 |  |  |  | 0 | T |
|  | 1 | 1 | 2 | 2 | 2 | 3 | 4 | 4 | 4 | 5 |  |  |  |  |  |
| patient 19 | 1 | 2 | 2 | 2 | 2 | 3 | 3 | 4 | 4 | 4 | 5 |  |  | 0.091 | T |
|  | 1 | 4 | 2 | 2 | 2 | 3 | 3 | 4 | 4 | 4 | 5 |  |  |  |  |
| patient 20 | 1 | 1 | 2 | 2 | 2 | 3 | 3 | 4 | 4 | 5 |  |  |  | 0.4 | T |
|  | 1 | 4 | 1 | 1 | 2 | 3 | 3 | 4 | 2 | 5 |  |  |  |  |  |
| patient 21 | 1 | 1 | 1 | 2 | 2 | 3 | 4 | 4 |  |  |  |  |  | 0.125 | T |
|  | 1 | 4 | 1 | 2 | 2 | 3 | 4 | 4 |  |  |  |  |  |  |  |
| patient 22 | 1 | 1 | 2 | 2 | 2 | 3 | 3 | 4 | 4 | 5 |  |  |  | 0.1 | - |
|  | 1 | 1 | 1 | 2 | 2 | 3 | 3 | 4 | 4 | 5 |  |  |  |  |  |
| patient 23 | 1 | 1 | 1 | 1 | 2 | 3 | 3 | 5 |  |  |  |  |  | 0.25 | - |
|  | 2 | 4 | 1 | 1 | 2 | 3 | 3 | 5 |  |  |  |  |  |  |  |

**S3 Table**. Confusion matrix for the perceptron classifier on healthy subjects’ components. Note that accuracy is defined for each class (network) as a matching rate to the viewer labels.

|  | | | | | |
| --- | --- | --- | --- | --- | --- |
|  | **Predicted Auditory** | **Predicted Visual** | **Predicted Default-mode** | **Predicted Motor** | **Accuracy** |
| Auditory | 35 | 0 | 0 | 3 | 92.1% |
| Visual | 0 | 46 | 2 | 0 | 95.8% |
| Default-mode | 1 | 4 | 64 | 3 | 88.9% |
| Motor | 5 | 2 | 4 | 83 | 88.3% |

**S4 Table**. Sensitivity, specificity and predictive values for each class (network) for the perceptron classifier on normal subjects’ components (for Viewer 1).

|  | Sensitivity | Specificity | Positive Predictive Value | Negative Predictive Value |
| --- | --- | --- | --- | --- |
| Auditory | 92.1% | 97.2% | 85.4% | 98.6% |
| Visual | 95.8% | 97.1% | 88.5% | 99.0 % |
| Default-mode | 88.9% | 96.7% | 91.4% | 95.6% |
| Motor | 88.3% | 96.2% | 92.3% | 93.3% |

**S5 Table**. Sensitivity, specificity and predictive values (average of Viewer 1 and 2) for each class (network) for the naïve Bayes classifier on epilepsy patients’ components.

|  | Sensitivity | Specificity | Positive Predictive Value | Negative Predictive Value |
| --- | --- | --- | --- | --- |
| Auditory | 90.9% | 98.1% | 78.3% | 95.1% |
| Visual | 100% | 100% | 96.6% | 99% |
| Default-mode | 88.9% | 96.4% | 86.2% | 96.2% |
| Motor | 78.6% | 91.3% | 85.6% | 94.4% |
| Exec. Control | 83.3% | 98.3% | 100% | 100% |

**S6 Table**. 18 temporal lobe epilepsy patient IC table and LOOCV errors for the Naïve Bayes classifier. Total accuracy = 86.2%. 1 – motor, 2 – default-mode, 3 – visual, 4 – auditory, 5 – executive control.

| **Patient #** | **Spatial map networks (top row) and classified output labels (bottom row)** | | | | | | | | | | | | | **Error** |
| --- | --- | --- | --- | --- | --- | --- | --- | --- | --- | --- | --- | --- | --- | --- |
| patient 1 | 1 | 1 | 1 | 2 | 2 | 3 | 3 | 4 | 4 | 5 |  |  |  | 0.4 |
|  | 2 | 4 | 1 | 2 | 2 | 3 | 3 | 1 | 1 | 5 |  |  |  |  |
| patient 2 | 1 | 1 | 2 | 3 | 3 | 5 |  |  |  |  |  |  |  | 0.167 |
|  | 1 | 1 | 2 | 3 | 3 | 1 |  |  |  |  |  |  |  |  |
| patient 3 | 1 | 1 | 1 | 1 | 2 | 2 | 2 | 3 | 3 | 3 | 4 | 4 | 5 | 0 |
|  | 1 | 1 | 1 | 1 | 2 | 2 | 2 | 3 | 3 | 3 | 4 | 4 | 5 |  |
| patient 4 | 1 | 1 | 1 | 2 | 3 | 3 | 4 | 5 |  |  |  |  |  | 0 |
|  | 1 | 1 | 1 | 2 | 3 | 3 | 4 | 5 |  |  |  |  |  |  |
| patient 6 | 1 | 1 | 2 | 3 | 4 | 4 | 4 |  |  |  |  |  |  | 0.143 |
|  | 1 | 3 | 2 | 3 | 4 | 4 | 4 |  |  |  |  |  |  |  |
| patient 7 | 1 | 1 | 2 | 3 | 3 | 4 | 4 | 5 |  |  |  |  |  | 0.125 |
|  | 1 | 1 | 2 | 3 | 3 | 1 | 4 | 5 |  |  |  |  |  |  |
| patient 8 | 1 | 1 | 1 | 3 | 3 |  |  |  |  |  |  |  |  | 0.4 |
|  | 1 | 2 | 4 | 3 | 3 |  |  |  |  |  |  |  |  |  |
| patient 9 | 1 | 1 | 2 | 3 | 3 | 4 | 4 | 4 | 5 |  |  |  |  | 0.333 |
|  | 2 | 4 | 2 | 3 | 3 | 4 | 4 | 4 | 1 |  |  |  |  |  |
| patient 10 | 1 | 1 | 1 | 2 | 3 | 3 | 4 | 4 | 5 |  |  |  |  | 0.222 |
|  | 2 | 1 | 1 | 2 | 3 | 3 | 4 | 4 | 1 |  |  |  |  |  |
| patient 11 | 1 | 1 | 1 | 1 | 2 | 2 | 3 | 3 | 4 | 4 |  |  |  | 0.2 |
|  | 1 | 1 | 2 | 1 | 4 | 2 | 3 | 3 | 4 | 4 |  |  |  |  |
| patient 12 | 1 | 1 | 2 | 2 | 3 | 3 | 4 |  |  |  |  |  |  | 0.143 |
|  | 2 | 1 | 2 | 2 | 3 | 3 | 4 |  |  |  |  |  |  |  |
| patient 13 | 1 | 1 | 1 | 2 | 2 | 3 | 3 | 4 | 4 | 5 |  |  |  | 0 |
|  | 1 | 1 | 1 | 2 | 2 | 3 | 3 | 4 | 4 | 5 |  |  |  |  |
| patient 14 | 1 | 1 | 1 | 2 | 2 | 2 | 3 | 3 | 4 | 5 |  |  |  | 0 |
|  | 1 | 1 | 1 | 2 | 2 | 2 | 3 | 3 | 4 | 5 |  |  |  |  |
| patient 16 | 1 | 1 | 1 | 1 | 1 | 2 | 2 | 3 | 3 | 3 | 4 | 4 | 5 | 0.077 |
|  | 1 | 1 | 4 | 1 | 1 | 2 | 2 | 3 | 3 | 3 | 4 | 4 | 5 |  |
| patient 17 | 1 | 1 | 2 | 3 | 3 | 5 |  |  |  |  |  |  |  | 0 |
|  | 1 | 1 | 2 | 3 | 3 | 5 |  |  |  |  |  |  |  |  |
| patient 18 | 1 | 1 | 2 | 2 | 2 | 3 | 4 | 4 | 4 | 5 |  |  |  | 0 |
|  | 1 | 1 | 2 | 2 | 2 | 3 | 4 | 4 | 4 | 5 |  |  |  |  |
| patient 20 | 1 | 1 | 2 | 2 | 2 | 3 | 3 | 4 | 4 | 5 |  |  |  | 0.3 |
|  | 1 | 4 | 1 | 1 | 2 | 3 | 3 | 4 | 4 | 5 |  |  |  |  |
| patient 21 | 1 | 1 | 1 | 2 | 2 | 3 | 4 | 4 |  |  |  |  |  | 0.125 |
|  | 1 | 4 | 1 | 2 | 2 | 3 | 4 | 4 |  |  |  |  |  |  |
